# A scoping review on natural cholesterol lowering supplements sold in South African pharmacies

**DOI:** 10.4102/hsag.v29i0.2299

**Published:** 2024-02-29

**Authors:** Hyeon Bok Lee, Razeeya Khan, Muhammed Vally, Ané Orchard

**Affiliations:** 1 Department of Pharmacy and Pharmacology, Division of Clinical Pharmacy, Faculty of Health Sciences, University of the Witwatersrand, Johannesburg, South Africa

**Keywords:** LDL, HDL, natural supplements, cholesterol, dyslipidaemia

## Abstract

**Background:**

Dyslipidaemia is defined as elevated total or low-density lipoprotein (LDL) levels or low levels of high-density lipoprotein (HDL). Patients may often make use of natural cholesterol lowering supplements (NCLSs) available at the pharmacy; however, limited information on these supplements is readily available. Pharmacists should be knowledgeable about NCLSs to ensure that the use of these supplements is supported by evidence and to provide appropriate advice to patients for desirable therapeutic outcomes.

**Aim:**

This study aimed to identify the NCLSs being sold in South African pharmacies and review the scientific evidence for each of the ingredients in these NCLSs.

**Methods:**

Seventeen NCLS products were identified, and the Joanna Briggs Institute (JBI) scoping review methodology was used to conduct a literature review of NCLSs.

**Results:**

From the ingredients reviewed it is evident that co-enzyme Q10, probiotics and sterols have sufficient evidence supporting their use. However, there is still limited scientific evidence available to validate the remaining ingredients.

**Conclusion:**

Further research on NCLSs will provide practising pharmacists and practitioners with a guide of the evidence available on the various ingredients in NCLSs.

**Contribution:**

This study provides a review of the available literature on the NCLSs being sold in the pharmacies across South Africa to provide pharmacists with a collated document of the evidence behind these popular supplements to assist them in making evidence based informed decision regarding natural products for cholesterol.

## Introduction

Cardiovascular diseases (CVDs) are a prominent leading cause of death worldwide, accounting for approximately 17.9 million deaths in 2019, which represents 32% of all deaths that year (World Health Organization [WHO] 2021). In South Africa, cardiovascular deaths account for nearly a fifth of total adult deaths (Statistics South Africa 2015). A close link between CVDs and dyslipidaemia exists (Carson et al. 2020). Although cholesterol is a vital biological molecule in the human body, excessive cholesterol is directly related to CVDs, and such levels are easily attained with an unhealthy diet (Lin, Lai & Kao 2015).

Cholesterol is transported in the blood by lipoproteins. The two main types of lipoproteins that carry cholesterol throughout the body are low-density lipoprotein (LDL) and high-density lipoprotein (HDL) (Lin et al. 2015). High-density lipoprotein is known as ‘good’ cholesterol, while LDL is known as ‘bad’ cholesterol (Elshourbagy, Meyers & Abdel-Meguid 2014). The HDL transports cholesterol to the liver, which is separated from the bloodstream before it builds up in arteries, while LDL transports cholesterol directly to arteries, this can result in atherosclerosis, a plaque buildup that contributes to CVDs (Goldstein & Brown 2015).

Dyslipidaemia is defined as elevated total or LDL cholesterol and triglyceride levels, and/or low levels of HDL cholesterol (Goldstein & Brown 2015). Dyslipidaemia can be present irrespective of race, sex, genes, genetic predisposition and age if lifestyle and dietary habits have deteriorated (Fodor 2010; García-Giustiniani & Stein 2016). The occurrence of cardiovascular events such as myocardial infarction or stroke is associated with low HDL levels and high LDL levels (Goldstein & Browns 2015). Patients who experienced a myocardial infarction were reported to have a significant increase in the cholesterol-carrying LDL and a reduced level of HDL (Goldstein & Browns 2015). Thus, controlling dyslipidaemia is essential in preventing and managing cardiovascular-related diseases.

Treatment of dyslipidaemia consists of lifestyle modification and drug treatment (American Heart Association [AHA] 2020). Although there is positive research and clinical trial data supporting the efficacy of cholesterol-lowering medications, patient compliance can be challenging because of the potential side effects or personal preference (Ward, Watts & Eckel 2019). Most cholesterol-lowering medicines decrease blood cholesterol levels with few side effects such as headache, dizziness and myalgia (Raal et al. 2011). Experienced by 5% to 10% of patients undergoing HMG-CoA reductase inhibitor therapy, also known as statin therapy, myalgia is one of the most frequently encountered side effects (Marais 2016). However, the type and severity of side effects vary from person to person (Raal et al. 2011).

Due to side effects and the desire to be on natural supplements, such as the complementary and alternative medicines, patients may seek an alternative treatment available at the pharmacy (Marais 2016). A case report of a 49-year-old man taking a statin for the past 3 years showed improved cholesterol levels and eventually reaching an acceptable cholesterol range. However, the patient continued to experience myalgia and a pharmacist advised discontinuing statin therapy and starting an over the counter product containing a natural supplement. After being on over-the-counter natural supplements for nearly 2 months, a significant increase in LDL was noted (Marais 2016). Recommending a natural cholesterol lowering supplement (NCLS) product with insufficient evidence can be harmful to a patient.

In accordance with the Good Pharmacy Practice (GPP) guidelines of South Africa, the well-being of the patient is the primary concern of a pharmacist (Good Pharmacy Practice in South Africa [GPPSA] 2017). Therefore, the objective of a pharmacist in providing medication therapy is to achieve appropriate therapeutic outcomes that enhance patient health and quality of life. A pharmacist cannot suggest an alternative treatment without consulting with the doctor when conventional treatments, which are prescribed substances, are available. When there is no scientific evidence of the effectiveness of supplements, a pharmacist must not give patients the impression that the product is effective (GPPSA 2017). Hence, a pharmacist must have thorough knowledge and provide impartial information to patients while being aware of the various types of non-conventional treatments available in the market.

Although NCLSs do not need to be regulated by Southern African Legal Information Institute ([SAFLII] 2020), pharmacists are responsible for knowing the evidence behind the products being sold at the pharmacy (GPPSA 2017). There is a critical need to review the evidence behind NCLS as pharmacists should be able to provide adequate information on what medications are safe and effective. However, because of limited information about NCLSs, limited advice is given to the patients (Fourie, Oosthuizen & Du Toit 2017).

This review aimed to evaluate the evidence behind the NCLS available in South African pharmacies and provide practising pharmacists and practitioners with a guide to the evidence available on the NCLS. The objectives were to identify and list the ingredients in the natural cholesterol-lowering supplements available in South African pharmacies, compare the ingredients in the health supplements against scientific literature available and determine whether ingredients effectively lower serum cholesterol levels, and lastly, to evaluate the claims made by the manufacturer regarding health supplement’s dosage, dosing regimen and its therapeutic effect of lowering cholesterol levels.

## Research methods and design

A selection of products claiming to reduce cholesterol or support the heart or heart health was collected from randomly chosen pharmacies in South Africa. The authors went into pharmacies and identified the cholesterol lowering supplements packed where the cholesterol supplements were stocked. Each supplement’s name was recorded, along with the ingredients, dosage and instructions. Product names were allocated pseudo names. A total of 17 NCLS products (50 ingredients) were identified in South African pharmacies (Table 1). The information on selected supplements was tabulated in Microsoft Excel 365. All ingredients mentioned in various supplements were reviewed and the article summary can be found in Online Appendix 1, Table S1 to Table S13.

**TABLE 1:** Investigative products with ingredients and dosage.

Product	Claim on the package	Ingredient	Strength	Dosage	Sufficient dosage‡
Product A	Cardiovascular support.	Co-enzyme Q10§	150 mg	Take 1 capsule daily, 2 h before supper.	120 mg
		Magnesium	150 mg		N/D†
		Vitamin D3	1000 IU		1000 IU
Product B	Reduces cholesterol.	Bergamot extract	200 mg	Take 1 tablet daily, at night.	N/D
		Plant sterols§	120 mg		1.5 g – 3 g
		Artichoke extract	80 mg		N/D
		Vitamin C§	20 mg		500 mg
Product C	Clinically proven to reduce cholesterol and inflammation without any known side effects.	*Lactobacillus Fermentum* ME3	8 billion CFU	Take 1 capsule daily.	6 billion CFU
Product D	Supports healthy blood sugar, blood pressure and blood lipid levels as well as overall general health.	Berberine HCI	500 mg	Take 2 capsules 3 times daily, before or with a meal.	N/D
		Origine 8®	25 mg		N/D
		Phytophare® green tea extract	15 mg		N/D
		Phosphatidylcholine complex	10 mg		N/D
		Chromium§	25 ug		N/D
Product E	Maintains and supports cardiovascular health.	Co-enzyme Q10§	150 mg	Take 1 capsule 1–2 times daily, preferably with a meal.	120 mg
		L-carnitine fumarate	100 mg		750 mg
		Vitamin C§	75 mg		500 mg
Product F	Heart support.	Co-enzyme Q10§	150 mg	Take 1 capsule daily.	120 mg
Product G	Contribute to the normal function of the heart.	Omega 3 fish oil	750 mg	Take 1 capsule daily.	500 mg
		EPA§	375 mg		N/D
		DHA§	45 mg		N/D
		Co-enzyme Q10§	10 mg		120 mg
Product H	Health supplement – name includes the word cholesterol and indicates it is for lowering cholesterol.	Vitamin A	2.27 mg	Take 1–4 sachets daily.	N/D
		Vitamin B6	50 mg		N/D
		Vitamin C§	3000 mg		500 mg
		Vitamin E (50%)	282 mg		N/D
		Folic acid§	400 mg		0.8 mg
		L-Arginine	200 mg		N/D
		L-Lysine	3000 mg		N/D
		L-Proline	500 mg		N/D
Product I	Help maintain homocysteine and cholesterol levels.	Betaine HCL§	250 mg	Take 1–2 tablets daily.	4 g
		Magnesium	100 mg		N/D
		Vitamin E acetate	14 IU		N/D
		Vitamin B2	12 mg		N/D
		Vitamin B6	12 mg		N/D
		Policosanol§	10 mg		10 mg
		Zinc	7.5 mg		N/D
		Folic acid§	0.2 mg		0.8 mg
		Vitamin B12	0.012 mg		N/D
Product J	Optimise health cholesterol and triglycerides.	Barberry root extract 60%	40 mg	Take 2 tablets 2–3 times daily, with meals.	10 g
		(*Berberis vulgaris*)	350 mg		459 mg
		Phytosterols§	150 mg		N/D
		Apple polyphenols 30% extract	12.6 mg		N/D
		Nicotinamide Co-enzyme Q10	15 mg		120 mg
Product K	Cholesterol support.	Phytosterols	400 mg	Take 2 tablets 1–3 times daily.	459 mg
		Co-enzyme Q10§	15 mg		120 mg
		Beta-carotene	195 ug		N/D
		Niacin (nicotinic acid)	5 mg		N/D
Product L	Cardiovascular health support.	Co-enzyme Q10§	150 mg	Take 1 capsule daily.	120 mg
		Bioperine	5 mg		N/D
Product M	Cholesterol support supplement.	Policosanol§	20 mg	Take 1 tablet daily, at night	10 mg
		Folic acid§	400 ug		0.8 mg
Product N	For the reduction of tiredness and fatigue. Although no claims related, it was with the cholesterol supplements.	Niacin (Vitamin B3)	35 mg	Take 1 tablet daily, after food.	N/D
Product O	Cardiac support.	Co-enzyme Q10	150 mg	Take 1 capsule daily.	120 mg
Product P	Controls cholesterol.	*Apium graveolens*	7 mg	Take 1–2 tablets 4 times daily.	N/D
		*Coriandrum sativum*	6 mg		N/D
		Purified *Ferula foetida*	7 mg		N/D
		*Cuminum cyminum*	7 mg		N/D
		*Embelia ribes*	10 mg		N/D
		*Piper longum*	15 mg		N/D
		*Zingiber officinale*	15 mg		N/D
		*Curcuma longa*	15 mg		N/D
		*Cyperus rotundus*	15 mg		250 mg
		*Allium sativum*	25 mg		N/D
		*Emblica officinalis* §	25 mg		500 mg
		*Terminalia belerica*	25 mg		25 mg
		*Terminalia chebula*	25 mg		25 mg
		*Bauhinia variegate*	25 mg		N/D
		*Plumbago zeylanica*	30 mg		N/D
		*Pterocarpus marsupium*	50 mg		N/D
Product Q	May support healthy cholesterol levels.	Nicotinamide (Vit B3)	16 mg	Take 1 tablet daily, after a meal.	N/D
		Beta-Phytosterols	150 mg		459 mg
		Inulin (*Fructooligosaccharides*)	200 mg		N/D
		Soy lecithin	20 mg		N/D

† , N/D, not defined;

‡ , sufficient dosage (a specific dosage that is deemed appropriate according to the scientific literature for achieving a therapeutic outcome based on the literature review);

§ , ingredients in NLCSs with substantial evidence.

The study was guided by the Joanna Briggs Institute ([JBI], 2015) scoping review methodology. This review aimed to provide descriptive evidence behind the NCLS available in South African pharmacies. This study design was chosen to allow the researcher to describe available evidence on NCLSs sold in South African pharmacies. The analysed papers were selected from three different electronic databases: PubMed, ScienceDirect and Scopus, accessed during the period 2020–2022. Additionally, unpublished literature obtained from Opengrey and Grey Literature Report was also utilised. The filters used included the terms ‘natural cholesterol supplement’, ‘cholesterol’, ‘lowering cholesterol’, ‘cholesterol supplement’, ‘triglycerides’, ‘LDL’, ‘HDL’ and the scientific or common name for each ingredient as listed in Table 1.

Inclusion criteria included the following:

1. Literature specific to the specific ingredients identified.
2. *In vitro* studies.
3. Animal studies.
4. Systemic reviews.
5. Meta-analysis.
6. All clinical trials.
7. Case reports.

Exclusion criteria included the following:

1. Lack of access to the complete paper after attempts were made to gain access.
2. If the article was not available in English.
3. Papers that were dated earlier than 2010, unless no review was available.

The researchers used a three-step search strategy to ensure a comprehensive search (The Joanna Briggs Institute 2015). The search strategy aimed to identify all included sources of information, both published and unpublished literature. The researchers searched for all sources of evidence simultaneously, which should result in greater sensitivity (The Joanna Briggs Institute 2015). The researcher made use of a reference manager, Mendeley, to assist with this process. The key information extracted during the study was recorded in a table. This included information related to the authors, year of publication, study method, outcome and interpretation. Titles and abstracts were screened for eligibility by two authors, and where disagreement existed, a third author was asked.

### Ethical considerations

This article followed all ethical standards for research without direct contact with human or animal subjects.

## Results

### Description of studies

#### Single ingredients

**FIGURE 1:**
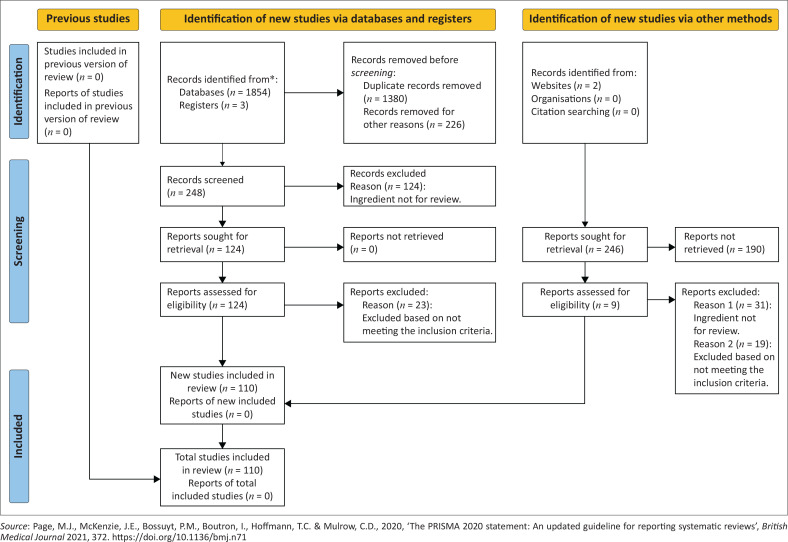
PRISMA diagram (Page et al. 2020): Summary of the flow of information in this review.

**FIGURE 2:**
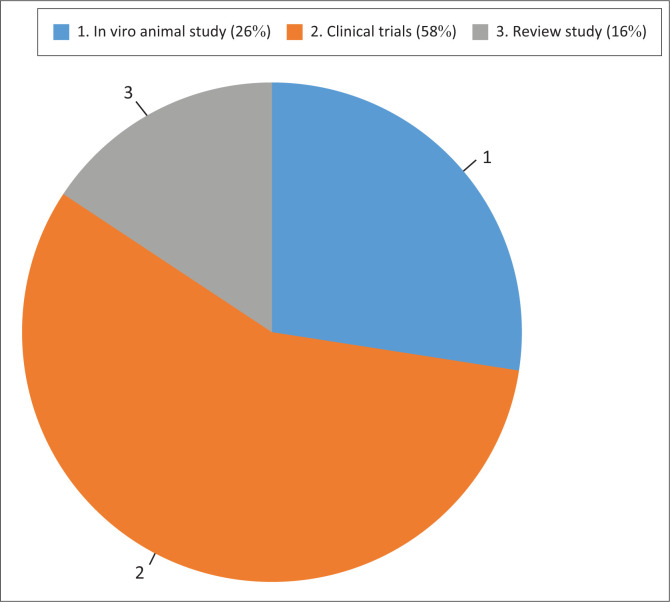
Overview of study types.

After the initial database search, 1854 articles were screened. Duplicates were removed, which reduced the number of articles countdown to 148, after which the abstracts were then screened, and additional articles were excluded based on not meeting the inclusion criteria. A total of 110 articles were read and reviewed. The process that was followed is summarised in the PRISMA diagram in Figure 1. Of the 110 articles, 28 were *in vitro/in vivo* studies, 64 were clinical trials and 18 were reviews (Figure 2).

#### Combination products

Articles containing multiple ingredients were reviewed, and a total of 24 articles were identified for this investigation. The reviewed article included ingredients not mentioned in the study, however, contains one or more ingredients from the current study as shown in Table S1 of Online Appendix 1.

### Categorising of scientific evidence

**FIGURE 3:**
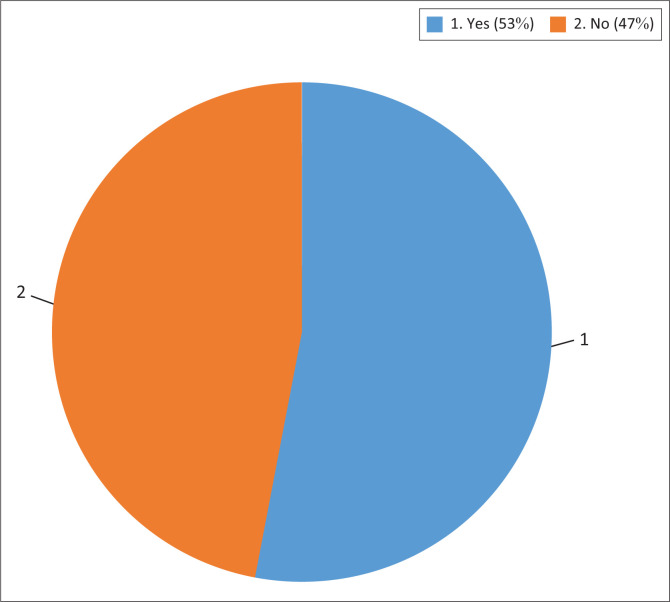
Article categorisation based on level of scientific evidence being sufficient.

Each article reviewed was categorised into either ‘yes’ or ‘no’ as illustrated in Figure 3, based on the level of evidence. The determination of the level of evidence for each study was based on the type of scientific evidence presented. Studies that included systematic reviews and meta-analyses of human trials were allocated a ‘yes’, while those that only included animal studies or human trials with limited evidence of beneficial effects were allocated a ‘no’. In all, 47% of the reviewed articles categorised as ‘no’, and 53% were categorised as ‘yes’.

## Discussion

**FIGURE 4:**
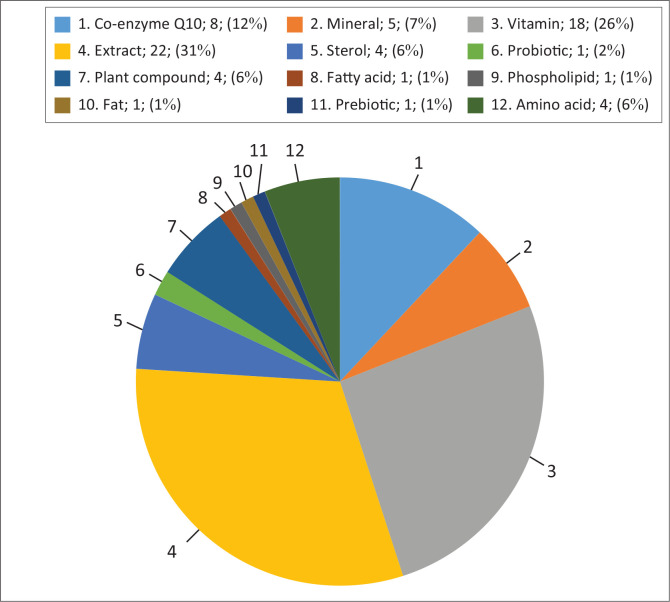
Graph summarising different classes of ingredients in various products (class of ingredient; number of counts; %).

**FIGURE 5:**
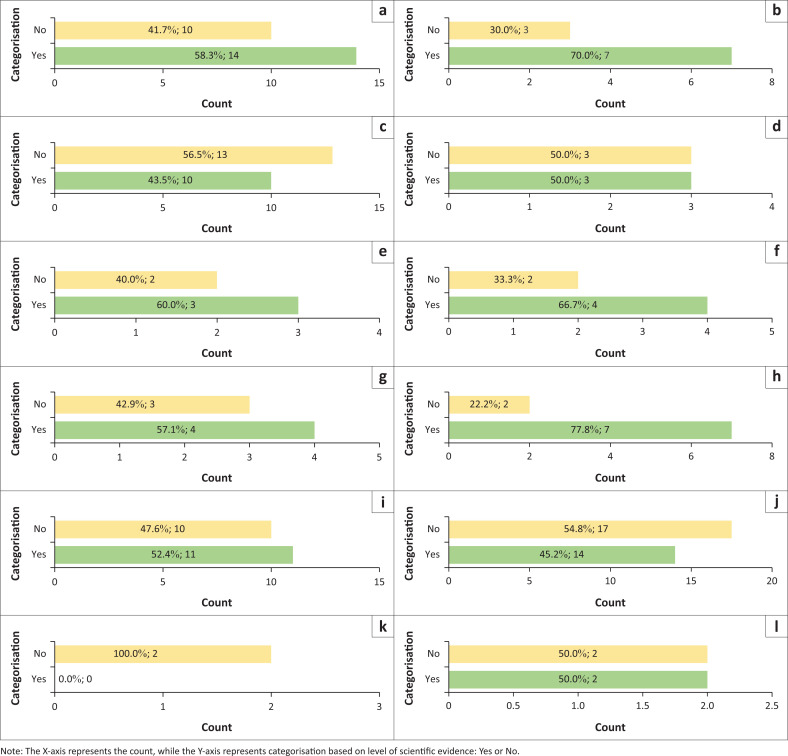
Summary of scientific evidence studied for each class of ingredients. (a) Combination. (b) Co enzyme Q10. (c) Vitamin. (d) Mineral. (e) Amino Acids. (f) Probiotics. (g) Fatty Acids. (h) Sterol. (i) Plant Compound. (j) Extract. (k) Phospholipids. (l) Fats.

From the 17 NCLSs products identified, a total of 50 ingredients were identified (see Table 1). Each ingredient was classified into different categories as summarised in Figure 4. Different classes of ingredients were then categorised into either ‘yes’ or ‘no’ based on the level of evidence in Figure 5. Of the 17 NCLSs products, 4 products had a single ingredient, while the rest were a combination of various ingredients. The most common types of ingredients found in the supplements were extracts with 22 different ingredients found in 5 different products, followed by vitamins with 10 different ingredients found in 10 different products. For the various ingredients, the minimum dose required for an effect was also tabulated (see Table 1).

A summary of scientific evidence studied for each class of ingredients is given in Figure 5. Of the 110 articles reviewed, 79 articles (53%) include systemic reviews and various randomised clinical trials to support its use as NCLS. However, not enough evidence is available to support its use as an alternative to statins. A total of 69 (47%) articles did not have sufficient scientific evidence to support the claims of cholesterol-lowering effects because of their study being a small-scale trial or being limited to *in vivo* studies.

### Co-enzyme Q 10

Co-enzyme Q10, most commonly identified in the NCLSs, is a vital substance needed for the correct functioning of the various organs and chemical reactions in the body. Aging and the use of statins lead to co-enzyme Q10 deficiency, and supplementation of co-enzyme Q10 may ease cardiovascular-related diseases (Aaseth, Alexander & Alehagen 2021). Co-enzyme Q10 has been widely used to assist in lowering cholesterol levels (Fan et al. 2017). It was noted that two products contained Co-enzyme Q10 as the only ingredient, and six products included Co-enzyme Q10 in combination with other ingredients. Co-enzyme Q10 was shown to have a beneficial effect in lowering cholesterol levels as well as decreasing the risk of CVDs at a dosage of 120 mg daily (Choi & Chae 2018; Fan et al. 2017; Jorat et al. 2018; Magno et al., 2018; Sabbatinelli et al. 2020; Tóth et al. 2017; Zhang et al., 2018a, 2018b). Additionally, when a co-enzyme Q10 is added to the statin therapy, the intensity of the statin side effects is significantly reduced (Qu et al. 2018).

### Vitamins

Vitamins were the second highest number of NCLSs after extracts. Vitamins are essential micronutrients that the body needs for adequate performance and well-being (Semba 2012). Vitamins are present in almost all foods; however, supplementation of certain vitamins such as vitamin D has a beneficial effect on reducing serum total cholesterol, LDL cholesterol and triglyceride levels at a dosage of 1000 IU – 1500 IU daily (Alves et al. 2021; Dibaba 2019; Holt et al. 2022; Tavakoli, Namakin & Zardast 2016; Yin et al. 2015). A combination of vitamin D (400 IU) and calcium (1000 mg) was beneficial in improving lipid profiles in postmenopausal women with dyslipidaemia (Schnatz et al. 2014). Of the 17 supplements investigated, a total of 9 products contained vitamins as a combination, and 1 product contained Niacin (vitamin B3) as the only ingredient. Niacin (1 g/day – 3 g/day) is beneficial in increasing HDL and reducing LDL, triglyceride and total cholesterol levels; however, possible side effects (flushing, liver damage) should be taken into consideration (Dwyer, Coates & Smith 2018; Julius 2015). Vitamin E on its own was not found to have any benefit on cholesterol. Studies of vitamin E combined with products such as omega-3 fatty acids were shown to decrease hypercholesteraemia (Taghizadeh et al. 2016). However, considering that omega-3 has been shown to be beneficial on its own (Blom et al. 2019; Oelrich, Dewell & Gardner 2013), it is doubtful that vitamin E was contributory. Added to that, vitamin E has been reported to increase the risk of haemorrhagic stroke (Schürks et al. 2010); therefore, its benefit in overall use in CVDs management should be cautioned. Vitamin C has been shown to decrease serum LDL and triglyceride levels (McRae et al. 2008), and folic acid has been shown to cause a decrease in risk of first stroke in patients with high total cholesterol (Qin et al. 2016).

### Extracts

The extracts derived from various plants were identified 22 times and seen in 5 different investigative products, making extracts the most common highest number of NCLSs identified on the South African market. Commonly used extracts such as garlic (*Allium sativum*) and red yeast rice (RYR) (*Monascus purpureus*) have been demonstrated to lower serum cholesterol (Barrat et al. 2013b; Zadhoush et al. 2021). Red yeast rice (RYR) is not included into investigative products; however, it is found through rice fermentation by the fungus *Monascus purpureus* and contains monacolin K, which is chemically identical to lovastatin and can cause the same types of side effects and drug interactions as lovastatin (Nguyen, Karl & Santini 2017). Red yeast rice containing 2 mg Monacolin K significantly reduces LDL in patients with unsatisfactory LDL (Minamizuka et al. 2021). The use of RYR can lead to gastrointestinal effects and potentially result in myopathy, hepatotoxicity, rhabdomyolysis and anaphylaxis, which are comparable to the adverse effects associated with statin usage (Nguyen et al. 2017). Lovastatin is a structurally similar derivative of simvastatin that is less potent, with a daily dose of 20 mg reducing cholesterol by 25% – 30% compared to 10 mg of simvastatin (Muscas, Louros & Osterweil 2019). Patients who supplemented with 800 mg and above of garlic showed a decrease in total cholesterol and LDL, as well as an increase in HDL (Alobaidi 2014; Aslani et al. 2016). For most extracts, inadequate evidence exists to support its scientific claims of hypolipidaemic effects. Importantly, the daily intake of extracts as a supplement can maximally reach tens of milligrams, which is far below the therapeutic dosage of the hypolipidaemic effect (Dwyer et al. 2018), thereby having little or no impact on the reduction of cholesterol levels (Ahmed Eid, Helal & Salah EL-Din Ahmed El-Wahsh 2011; Alobaidi 2014; Al-Azhary 2011; Arnaboldi, Corsini & Bellosta 2022; Bao, Bai & Borijihan 2012; Barrat et al. 2013a, 2013b; Bhandari et al. 2013; Chew et al. 2019; Cicero et al. 2019; Donato et al. 2021; Ghyasi, Mohaddes & Naderi 2019; Iskandar et al. 2020; Koutsos et al. 2020; Maruthupandian, Maruthupandian & Mohan 2011; Moelviani and Armansyah 2019; Mohammadi et al. 2013; Ogier et al. 2013; Okwu et al. 2015; Pendurkar & Mengi 2009; Riva et al. 2021; Rondanelli et al. 2013; Ruscica et al. 2019; Setyowati & Ulya 2021; Tripathi, Gupta & Singh 2019; Uchendu et al. 2020; Upadya et al. 2019; Whitfield et al. 2016; Yang et al. 2018; Zadhoush et al. 2021; Zeb et al. 2018). Despite the scientific claims of hypolipidaemic effects associated with most extracts, there is insufficient evidence to support these claims. The recommended daily intake of these extracts was not more than tens of milligrams, which is less than the therapeutic dose recommended to induce a hypolipidaemic effect. Therefore, taking these extracts as a supplement is unlikely to have a significant impact on reducing cholesterol levels.

### Sterols

‘Plant stanols and sterols, also known as phytosterols, are cholesterol-like compounds that are found naturally in a range of plant-based foods’ (BDA 2021, viewed 13 July 2022). The cholesterol lowering effect of phytosterols is recognised to result from the inhibition of intestinal cholesterol absorption (Trautwein et al. 2018). Daily intake of phytosterols alone at 1.5 g – 3 g daily lowers LDL-cholesterol and triglyceride by 7.5% to 12% and 8.3%, respectively (Blom et al. 2019; Demonty et al. 2013; Han et al. 2016; Smet, Mensink & Plat 2012; Trautwein et al. 2018). A study by Han et al. (2016) found that supplementation of 2.5 g daily of phytosterols in patients treated with statins led to a significant decrease in LDL-cholesterol and total cholesterol levels by 0.30 mmol/L each, compared to statins alone. However, the impact of phytosterols on preventing CVD lacks randomised data according to the same study. The LDL cholesterol is a known risk factor for arteriosclerotic CVD and a reduction in LDL-C concentration can be achievable with phytosterols intake (Ference et al. 2017). Based on the evidence, in patients that do not respond adequately to single statin therapy, stanols or sterols can be added as a complementary medicine with low statin doses to avoid possible adverse effects (Párraga-Martínez et al. 2015).

### Probiotics

Probiotics are live micro-organisms intended for health benefits when consumed. *Lactobacillus fermentum* ME-3 is a unique strain of *Lactobacillus* species, having health benefits, including cholesterol-lowering effects on humans and lowering the risk of CVDs by reducing the formation of oxidised LDL and triglyceride when 8 billion Colony forming units (CFUs) of *Lactobacillus fermentum* ME-3 is consumed (Mikelsaar et al. 2015; Wang et al. 2018). Probiotics promote LDL-C and triglyceride reduction by reducing the intestinal cholesterol absorption (Magno, Ceccarini & Pelosini 2018). Probiotics such as *Lactobacillus* species, when used as a supplement, were found to be effective from a dose of 5 billion CFUs or higher (Kligler & Cohrssen 2008). Supplementation of *L. fermentum* ME-3 may also help to prevent risk, alleviate the symptoms and treat metabolic-related conditions such as diabetes (Kullisaar et al. 2016). Only one product on the South African market contained 8 billion CFUs of *L. fermentum* ME-3 as a single ingredient, which is sufficient to have cholesterol-lowering effects.

### Plant compounds

Berberine and policosanol are two bioactive compounds that fall under this category. Berberine, which can be extracted from various plants, has been found to have lipid-lowering and insulin-resistance properties at a dose greater than 500 mg daily (Investigative product D) (Cicero & Baggioni 2016; Hu et al. 2012; Koppen et al. 2017; Spigoni et al. 2017; Zhao et al. 2021). When taken alone or in combination with other ingredients, berberine has been shown to lower average LDL cholesterol levels by 20% – 30%. Policosanol, on the other hand, is a sugar cane extract that contains aliphatic alcohols that assist in downregulating cholesterol synthesis (Park et al. 2019). A study conducted in Cuba found that a daily dose of 10 mg of policosanol led to a significant reduction in total cholesterol, LDL and triglycerides, as well as an increase in HDL (Park et al. 2019). There is not sufficient research demonstrating a benefit by betaine to lower cholesterol; however, it may be beneficial for lowering homocysteine (Albuquerque et al. 2017; Ashtary-Larky et al. 2021; Dong et al. 2021; Grizales et al. 2018).

### Fatty acid (Omega-3)

Omega-3 fatty acids are a group of polyunsaturated fatty acids found in seafood that is required for numerous functions in the body. A study suggests that the long-chain fatty acids such as eicosapentaenoic acid (EPA) and docosahexaenoic acid (DHA) can reduce blood triglyceride levels (Peters et al. 2012). The recommended daily dosage of EPA and DHA for heart-healthy diet is 250 mg – 500 mg daily; however, no strong evidence on the protective effect against the heart disease is available (Choi & Chae 2018; Peters et al. 2012; Siscovick et al. 2017). The supplementation of Omega-3 increases the levels of LDL and may also increase the risk of prostate cancer; therefore whether Omega-3 supplements are beneficial is uncertain (Tóth et al. 2017). Thus, statin and omega-3 fatty acid combination should be cautiously recommended, considering the safety issues associated with its use.

### Soy

The cholesterol-lowering effect of soy is well known and led to regulatory approval of a health claim, by the U.S. Food & Drug Administration, relating to soy protein reducing the risk of CVDs (Ramdath et al. 2017). However, soy contains additional ingredients such as isoflavones, lecithins, saponins and fibres that may improve cardiovascular health through separate mechanisms (Ramdath et al. 2017). The supplementation of soy food products (25 g) decreased the total and LDL cholesterol by 0.23 mmol/L and 0.18 mmol/L, respectively (Jung et al. 2021). No scientific evidence was available for its beneficial effect as a combination with statins or other ingredients.

### Prebiotic (Inulin [Fructooligosaccharides])

Prebiotics are non-digestible food components that selectively stimulate the growth or activity of desirable microorganisms (probiotics). Inulin is a carbohydrate belonging to a class of compounds known as fructans and is known to lower serum cholesterol at a daily dosage of more than 2 g (Kassaian et al. 2019). However, from the scientific evidence reviewed, there were no significant effects of the prebiotic supplementation on serum total cholesterol, LDL and HDL (Taghizadeh et al. 2014); thus their only benefit would be promoting the growth of probiotics.

### Amino acids

Amino acids are referred to as the building block proteins. The amino acid L-arginine has received much attention because of its numerous beneficial effects of significantly improving lipid profiles (Hadi et al. 2019). However, L-arginine supplementation has not been proven to significantly change the concentrations of total cholesterol or HDL (Hadi et al. 2019). The study conducted by Hadi et al. (2019) found that there was only a decrease in triglyceride levels, which suggests inadequate evidence to support the cholesterol-lowering effects of the amino acids. In general, amino acids are consumed alongside various other components, often including vitamins found in investigative product H.

## Overview

The beneficial role of NCLS can be seen in several studies; however, caution should be exercised by those still deficient in clinical studies. In general, any food has the potential to be used as a drug or supplement depending on the content of its active compounds. The finding of the current review showed that supplementation of sterols, co-enzyme Q10 and probiotics can improve lipid profiles and reduce statin-related side effects. According to a meta-analysis of 12 randomised controlled trials, patients taking statins who supplemented with co-enzyme Q10 experienced a significant reduction in the incidence of muscle pain (Bookstaver, Burkhalter & Hatzigeorgiou 2012; Kumar et al. 2012). A meta-analysis of 15 randomised clinical trials, involving over 500 participants, has demonstrated the effectiveness of sterol supplementation in neutralising the intestinal absorption of cholesterol caused by statin therapy (Poli et al. 2021). Consuming 8 billion CFUs of *L. fermentum* ME-3 probiotic has been found to help decrease the production of oxidised LDL and triglycerides by decreasing the absorption of cholesterol in the intestines (Mikelsaar et al. 2015; Wang et al. 2018).

Of the 17 NCLS products identified and investigated, 13 were a combination of multiple ingredients. The products with multiple ingredients may influence the cholesterol levels directly or indirectly by triggering more than one mechanism. However, considering the evidence of these products in combination is limited; the effect of the combinations is unknown. Added to that, if one considers the effects of natural product interaction studies in general (Hübsch et al. 2014; Orchard et al. 2018), it is clear that not all combinations would result in an advantageous outcome and certain ingredients may have the potential to negate each other’s effects, and as there is insufficient evidence regarding their combination, the effectiveness of these supplements cannot be conclusively determined. Synergy cannot be assumed despite the benefits of each product proven as a single entity. Products in combination need to be studied in the combination they are in to establish what the interactive profiles of the combined products would be, thus caution and a need for scientific evidence of the combination NCLSs is strongly recommended.

### Side effects and drug interactions

Those who are taking NCLS should be advised to have regular follow-ups with a medical professional to monitor for potential side effects and interactions with ingredients from the supplements and drugs. Common ingredients such as *Allium sativum* (Garlic), green tea extract, policosanol and fish oil should be taken with care as they may interact with some blood-thinning medications (Zadhoush et al. 2021). Common gastrointestinal side effects such as diarrhoea, nausea and vomiting can be experienced in ingredients such as berberine, fish oil and green tea extracts (Zhao et al. 2021). Red yeast rice is used as a dietary supplement to lower cholesterol levels; however, it should not be taken with statins, because it may enhance their effect and increase the risk of liver damage (Farkouh & Baumgärtel 2019), which should be considered for studies to determine whether this may be a beneficial combination to decrease the statin dose.

## Conclusion

After reviewing 50 ingredients, sterols (with 77% of sufficient scientific evidence), co-enzyme Q10 (70%) and probiotics (*L. fermentum* ME-3) (66%) were noted as having evidence demonstrating cholesterol lowering effects when taken at the correct dosage. Different factors need to be considered, as some ingredients may cause side effects or may interact with other medications leading to undesirable therapeutic outcomes.

The limited scientific evidence on certain ingredients, having one study or several small studies with no comparison group, may lead the products to be less known to the public. Despite some of the ingredients containing sufficient scientific evidence if used alone, studies on the combinations of majority of the ingredients are lacking. Added to that, the dosage required for pharmacological activity needs to be emphasised in studies, and these further need to be taken into consideration for the NCLSs.

Pharmacists play a crucial role in recommending and advising on the use of NCLS. However, it is essential for them to exercise caution and thorough evaluation when considering the recommendation of such products. This evaluation should involve assessing the available evidence regarding the ingredients used in the NCLS product, determining the minimum effective dose required for the desired effects and being mindful of potential side effects.

Pharmacists have a professional responsibility to facilitate informed decision-making on the use of NCLS based on scientific evidence, promoting patient safety.
